# Tribological investigation of diamond-like carbon coated micro-dimpled surface under bovine serum and osteoarthritis oriented synovial fluid

**DOI:** 10.1088/1468-6996/16/3/035002

**Published:** 2015-05-14

**Authors:** Subir Ghosh, Dipankar Choudhury, Taposh Roy, Azuddin Bin Mamat, H H Masjuki, Belinda Pingguan-Murphy

**Affiliations:** 1 Department of Biomedical Engineering, Faculty of Engineering, University of Malaya, 50603, Kuala Lumpur, Malaysia; 2 Faculty of Mechanical Engineering, Brno University of Technology, Technická 2896/2, 616 69 Brno, Czech Republic; 3 Central European Institute of Technology, Brno University of Technology, Technická 3058/10, 616 00 Brno, Czech Republic; 4 Department of Mechanical Engineering, University of Malaya, 50603, Kuala Lumpur, Malaysia

**Keywords:** friction, wear, DLC, dimple, OA synovial fluid

## Abstract

Osteoarthritis-oriented synovial fluid (OASF), i.e., that typical of a patient with osteoarthritis, has different physical and biological characteristics than bovine serum (BS), a lubricant widely used in biotribological investigations. Micro-dimpled and diamond-like carbon- (DLC) coated surfaces are key emerging interfaces for orthopedic implants. In this study, tribological performances of dimpled surfaces, with and without DLC coating, have been investigated under both BS and OASF. The friction tests were performed utilizing a pin on a disk tribometer, whereas contact pressure, speed, and temperature were simulated to a ‘medium walking gait’ of hip joint conditions. The mechanical properties of the specimen and the physical properties of the lubricant were characterized before the friction test. Raman analysis was conducted to identify the coating condition both before and after the test. The DLC-coated dimpled surface showed maximum hardness and residual stress. A DLC-coated dimpled surface under an OASF lubricated condition yielded a lower friction coefficient and wear compared to those of plain and dimpled specimens. The higher graphitization of coated materials with increasing load was confirmed by Raman spectroscopy.

## 1. Introduction

Improvements in medicine and healthcare have increased life expectancy, especially among aging people for the last six to 10 decades [1]. However, elderly people are more likely to suffer from osteoarthritis (OA); subsequently, the high demand for joint replacement has become yet higher [2]. Although hip replacement is considered one of the most successful orthopedic surgical procedures, satisfaction has not been achieved yet, especially as recent statistics showed that the failure rate of hip joints is around 15% within 15 years of their implantation [3]. Therefore, a number of studies have been carried out based on different materials and designs to increase the durability of implants by decreasing friction, wear, and corrosion. The bearing clearance, which is the difference between the radius of the prosthesis head and cup, is an important factor of hip joint-bearing performance and stability [4, 5]. The recommended clearance of a modern hip joint prosthesis is usually 50–100 microns [6, 7]; however, it is a big challenge for manufacturers to maintain the clearance with an exact deviation, despite the availability of precise manufacturing facilities. Furthermore, the clearance is a variable parameter over time and mode of sliding. Therefore, a super wear-resistant material along with a lower friction yielding capability is desirable for the hip joints’ interface.

Ti–6Al–4V alloy is commonly used in biomedical, aerospace, and automotive industries because of its excellent mechanical and physical properties, such as high specific strength, high melting point, high corrosion resistance, and bioactivity [8]. However, it exhibits poor tribological performance, leading to research aiming to improve its tribological performance by surface modification techniques [9, 10]. One effective technique to do this is fabricating micro-dimples on a Ti–6Al–4V alloy substrate. It was reported previously that micro-dimples exhibit a low coefficient of friction [11, 12] and wear rate [13, 14] because they improve lubrication, trap wear debris generated from the contacting surface, and provide a hydrodynamic pressure [5]. Thus, surface texturing, such as the use of micro-dimples, enhances tribological performance in the presence of a lubricant. However, micro-dimples may rapidly wear out after an extended period of cyclic loading. Such loading typically includes a number of different lubrication modes; for example, near to the resting period of a gait, dimples becomes less effective in forming lubrication film thickness because of the low speed and high pressure. In these circumstances, a boundary or a solid lubricant can play a vital role. Diamond-like carbon (DLC) films are considered one of the best solid lubricants and have a high wear resistance capability. For example, DLC acts as a solid lubricant by transforming into graphite where it builds up a transfer layer on its counterpart [15, 16], so it causes an easy slip between the contact surfaces. Furthermore, it has been popular due to its biocompatibility, hardness, wettability, corrosion resistance properties [17, 18], and suitability of fabrication in a complex shaped specimen. Amanov and Sasaki [8] revealed a significant reduction of friction and wear rate by utilizing a combination of DLC-coated micro-dimple on a Ti–6Al–4V substrate under oil-lubricated sliding conditions. Very recently, Choudhury *et al* [3] revealed that DLC-coated micro-dimples have dual benefits: The dimples provide a thicker film thickness, and DLC enhances boundary lubrication. However, in that study, only bovine serum (BS) was used, and the conditions of post-experimental DLC were not comprehensively studied. Therefore, it is still not clear if the tribological behavior of DLC-coated micro-dimples works under body fluids, which have similar properties to OASF. The status of the coating after extended cyclic loading is also unclear.

In order to establish likely *in vivo* tribological outcomes, an appropriate lubrication of joint interfaces should be utilized *in vitro*. Normally, researchers use water or BS in their *in vitro* testing; however, such fluids do not accurately simulate body fluids [19, 20]. Because BS does not have most of the tribologically active proteins, such as (globulin, lubricin) and hyaluronic acid (HA), it is therefore very difficult to predict the influence of body fluids on the friction and wear mechanism. Moreover, the fundamental lubrication mechanism between natural joints and OA-affected joints is different, since the effects of the protein—surface interactions play a significant role [21, 22]. It is crucial to understand what is happening in the actual contact zone of artificial joints. It is also important to understand *in vivo* lubrication mechanisms under a lubricant that matches the physiological conditions of an OA patient. This study may posit better suggestions for component design and implantation. To our knowledge, there is no study conducted with OASF (considering all major biological components with an appropriate concentration ratio) in tribological investigation on the advanced interface of hip joints. A successful DLC-coated micro-dimple interface can offer a single part of the stem and head without having a tapper joint, which could solve the problem of fretting wear. Therefore, the objective of this study is to investigate the tribological influence of a dimpled surface and a DLC-coated dimpled surface under BS and OASF conditions (a defined composition).

## 2. Materials and methods

### 2.1. Sample preparation

According to the tribometer (TR 283 Series, DUCOM, Bangalore, India) dimension, both disk and pin (rod) were prepared from commercially obtained titanium grade 5 alloy (Nova Scientific, Malaysia), commonly known as Ti-6Al-4V. The disk and pin were prepared in their respective dimensions: 15(L) mm × 15(W) mm × 6(H) mm and 6(L) mm × 6(∅) mm. Each disk went through a series of polishing processes using various grades of silicon carbide paper: 1000, 1200, 1500, and 2000, and finally with a diamond polycrystalline suspension (0.02 micron) on a polishing cloth. In this experiment, three types of samples were prepared, namely, plain surface (T_1_), dimpled surface (T_2_), and DLC-coated dimpled surface (T_3_).

### 2.2. Surface modification

#### 2.2.1. Dimple fabrication

Briefly, the CATIA V5 design software was used to draw the dimple array patterns. A CNC micro drilling machine (Mikrotools DT110, Singapore) was used to create the micro-dimples in a circular shape. (This is advantageous, as it can be precisely controlled and applied to curved surfaces without any significant change in bulk material properties.) Our previous study also confirmed that the possibility of the presence of wear debris from a drill bit is markedly reduced and thus also suitable for biomedical applications [12, 14]. A diamond drill bit (UKAM Industrial Superhard Tools, US) with a diameter of 400 *μ*m was used to fabricate dimples on titanium alloy disks.

#### 2.2.2. Coating deposition

In the current research, dimpled surfaces were coated using a physical vapor deposition process. The machine (Milman Hybrid Decocoater) was equipped with an integrated arc and magnetron sputtering technology for greater flexibility to deposit hydrogenated amorphous carbon (a-C:H) DLC [23]. The DLC coating on dimpled titanium alloy disks was performed through five steps, namely, evacuating, argon cleaning, chromium deposition, DLC deposition, and, finally, cooling. First, the samples were degreased, chemically etched, and then put into a deposition chamber where samples were subsequently evacuated to a vacuum of 2 × 10^−5^ mbar. Second, argon cleaning was conducted. Third, chromium was deposited for 10 min as an interlayer. After that, the amorphous carbon a-C:H was deposited for 90 min, where coating thickness was controlled by deposition time. Finally, samples were cooled for 30 min.

### 2.3. Lubricants preparation

#### 2.3.1. Osteoarthritis-oriented synovial fluid (OASF)

The OASF lubricant composition is summarized in table 1. The formulation is based on the concentration and composition of an OA patient’s synovial fluid [20, 24, 25]. The powder- form composition was dissolved in a phosphate buffered solution (PBS) at concentrations as stated in table 1 to prepare OASF that represents body fluid affected by OA. Mucin, type III was used, which is closest to lubricin protein, as it is difficult to extract lubricin through the many steps of the purification process.

**Table 1.** The composition and concentration of OASF.

Product Name	Product Code	Solvent	Final concentration (mg ml^−1^)
Hyaluronic Acid (HA)	MP Biomedicals, USA #0215993350		2.5
Bovine albumin	MP Biomedicals, USA #0332		18.1
Bovine *γ*-globulin	Sigma, US #G5009	PBS^a^	13.1
Mucin, Type III	Sigma, US #M1778		0.2

a Phosphate buffered solution (PBS) (Sigma-Aldrich # 4417)

#### 2.3.2. Bovine serum (BS)

The BS (HCL#SV30160.03; HyClone Fetal Bovine Serum) was obtained commercially from Life Technologies (Sigma-Aldrich #4417). The control lubricant is made up of 30% BS with 70% distilled water. All lubricants were stored at −20 °C, and, prior to the friction test, the lubricants were kept in an oven at 37 °C for 1 h to achieve normal body temperature.

### 2.4. Characterization

#### 2.4.1. Dimple profile

**Figure 1.**
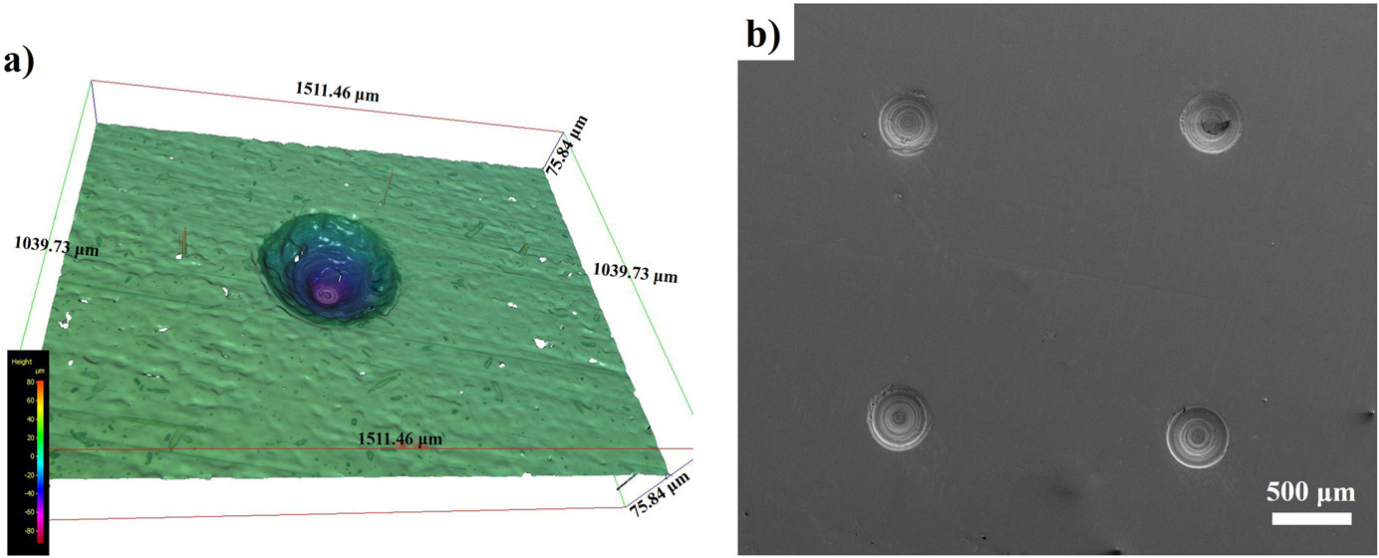
(a) Structure of a micro-dimple produced by 3D optical profiler and (b) FESEM image of dimpled surface.

The dimple array patterns were observed by field emission scanning electron microscopy (FESEM), (AURIGA, Zeiss Singapore). A three-dimensional (3D) optical profiler (Alicona Infinite Focus, Chicago, USA) was used to measure diameter, pitch, and depth of the dimples. The structure of the micro-dimple shown in figure 1(a) was produced by a 3D optical profiler. Figure 1(b) shows the morphology of the dimpled surface as measured by FESEM.

#### 2.4.2. Coating characterization

**Figure 2.**
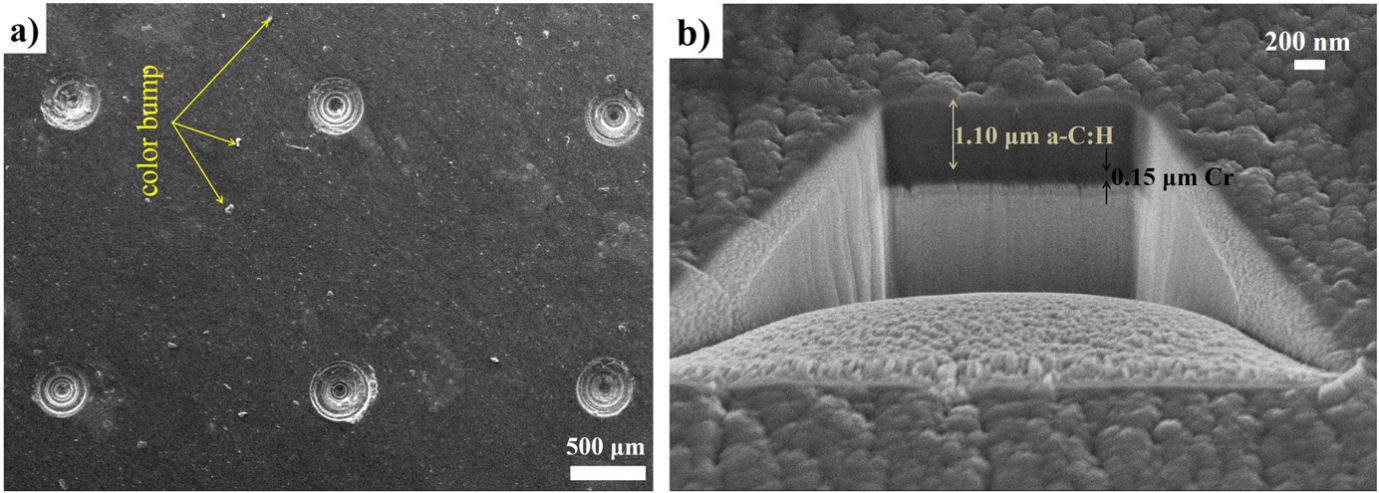
(a) SEM image of DLC-coated dimpled surface and (b) coating thickness profile of a-C:H.

FESEM was used to observe the DLC-coated dimpled surface (figure 2(a)) and to measure the thickness of the coating (figure 2(b)). The coating thickness (1.10 ± 0.05 *μ*m) was measured by a focused ion beam technique. The highly energized ion beam was charged on the coated surface to dig a groove.

#### 2.4.3. Mechanical properties

A 3D optical profiler was applied for surface roughness measurement on different surface conditions T_1_, T_2_, and T_3_. The hardness of each sample was measured before and after coating. In general, three non-dimpled areas were selected for measurement. For samples T_2_ and T_3_, three different zones were selected correspondingly near the edge of a dimple, 50 *μ*m from the edge of a dimple, and 100 *μ*m from the edge of a dimple. A dynamic ultra-micro hardness tester (Shimadzu DUH-211/DUH-211S) was used to measure the hardness. In this study, residual stress was calculated for both dimpled (T_2_) and DLC-coated dimpled surfaces (T_3_) on dimpled areas using an x-ray diffractometer (XRD) (PANalytical Empyrean). The XRD-sin^2^*ψ* technique was applied using CuK*α* radiation (0.154 0598 nm) at 40 kV and 40 mA. Tilt angles (*ψ*) were applied ranging from 0–35° by a computer-controlled Omega-goniometer. Each measurement was repeated five times for accuracy.

#### 2.4.4. Physical properties measurement

Prior to the friction test, the viscosity of the lubricants was measured by using a Brookfield Viscometer –LV (DV-11 + Pro EXTRA) at a 25 °C temperature at a definite shear rate 100 s^−1^.

Wettability measurement reveals the information about surface energy of the material and the interaction of the coated surface with lubricants in terms of contact angle. A contact angle analyzer (OCA15EC, Data Physics Instruments, Germany) was used to measure the contact angle. The static sessile drop method was applied, where 2 *μ*l lubricants were dropped through a syringe onto a substrate. The three types of sample (T_1_, T_2_, and T_3_) were subjected to a wettability test with three types of lubricants, namely water, BS, and OASF. Each measurement was repeated five times for better accuracy.

### 2.5. Friction test

The samples, namely T_1_, T_2_, and T_3_, were subjected to a friction test. A tribometer (TR 283 Series, DUCOM, Bangalore, India) was used in this experiment. The schematic diagram of the experimental setup has been shown in previous studies [12, 14]. Loads of 10, 15, and 20 N were used, corresponding to mean Hertzian contact pressures of 107 MPa, 131 MPa, and 151 MPa, respectively. Pressures in a diarthrodial joint are usually around 5 MPa and can be as high as 18 MPa [26]. However, it is higher for artificial implants, because Hertzian contact pressure depends on the modulus of elasticity of the material, contact area, and applied load. Our estimated contact pressure was kept as low as possible and was lower compared to previous studies [27, 28]. Total running time for each loading condition was 90 min. Table 2 summarizes the experimental setup of tribology testing. Parameters were selected based on simulated hip joints conditions [12, 27].

**Table 2.** Experimental parameters.

Items	Description
Specification of pin	Diameter—6 mm, Length—6 mm
Specification of disk	L—15 mm, W—15 mm, H—6 mm
Speed	20 mm s^−1^
Hertz contact pressure	107 MPa, 131 MPa, 151 MPa
Temperature	37 °C

### 2.6. Raman analysis

A Raman microscope is commonly used to characterize the structural changes of coated samples due to friction [29]. In our study, Raman analysis was carried out by using an Ar^+^ laser operating at 514.5 nm, and Raman spectra were taken from 800–2400 cm^−1^. The laser beam was focused to a 2 mm spot size, and Raman spectra of the coatings were taken in spot mode. The Raman analysis was only conducted for T_3_ samples, and this experiment was repeated after the T_3_ sample had undergone friction tests. The Raman analysis was focused on the as-deposited surface condition and wear track region after friction tests for different loads.

### 2.7. Wear analysis

The weight of the pin and disk was measured both before and after friction tests by a digital balance (Oertling VA304) than can measure up to 0.010 ± 0.005 mg to retrieve weight loss due to friction. Thus, the wear rate of the pin and disk was calculated from the weight loss of the sample. After friction tests, samples were dried to confirm no weight gain due to lubricant contamination. Also, the surface morphologies of worn surfaces after friction tests were analyzed by FESEM.

### 2.8. Statistical analysis

Statistical analysis was performed by the IBM SPSS statistics 21 software to identify whether there is any significant difference in friction coefficient value for the different lubricants. For this purpose, a two-way analysis of variance was performed on all subsets of data in each study to compare between lubricants over time.

## 3. Results and discussion

### 3.1. Dimple measurement

The dimple array is an important parameter for better lubrication in artificial joint prostheses. Recent studies revealed that the dimple diameter, density, and depth have major effects on the friction coefficient value [12, 30]. In our study, the dimple parameters are similar for two surface conditions, T_2_ and T_3_, as measured by a 3D optical profiler. The dimple parameter is presented in table 3. The small standard deviation confirms the accuracy of producing micro-dimples by a diamond drill bit. It also shows that there is no significant difference in depth of dimple due to DLC coating, because coating thickness was very low, at 1.10 *μ*m on average.

**Table 3.** Dimple parameters on two surface conditions (± shows the standard deviation of measured value).

Samples	Diameter, ∅ (*μ*m)	Depth, h (*μ*m)	Aspect ratio, *λ* = h/∅	Pitch (*μ*m)	Dimple density, A (%)	Total no. of dimples
Dimpled surface (T_2_)	410 ± 5	30 ± 2	0.073	950 ± 20	16.20	64
DLC-coated dimpled surface (T_3_)	410 ± 5	30 ± 2	0.073	950 ± 20	16.20	64

### 3.2. Coating characterization

The FESEM image shows the morphology of the DLC-coated dimpled surface at deposited condition (figure 2(a)). Carbon should be the main composition on the coating layer; it also contains chromium, as it was used as an interlayer during the deposition process in order to increase the bonding strength between coating and titanium alloy substrate [5]. Furthermore, some pores, as well as lighter-colored bumps, may exist on the coating surfaces (figure 2(a)). The coating thickness was 1.05–1.15 *μ*m for DLC coating (figure 2(b)). Previous studies on DLC coating also showed a similar coating thickness value [5, 23].

### 3.3. Mechanical properties

The mirror-polished surface is desirable to understand the effect of lubrication in tribology. Higher values of surface roughness have an adverse effect on tribological performance. Our study represents a standard surface roughness profile, which is comparatively lower than previous studies [19, 31]. The lower surface roughness was achieved by multistage polishing of the titanium substrate. The mirror-polished surface was obtained by removing sharp edges from the substrate. Titanium alloy is comparatively softer and easier to polish to a mirror surface. The mechanical properties of all samples are presented in table 4.

**Table 4.** The mechanical properties of the material at different surface conditions.

Surface condition	Surface roughness (nm)	Hardness (HV)	Residual stress (MPa)
Plain surface (T_1_)	50 ± 5	326	—
Dimpled surface (T_2_)	50 ± 5	325	245
DLC-coated dimpled surface (T_3_)	50 ± 5	698	477

DLC coating increases the hardness of a substrate material and plays a significant role in friction [32]. It enhances the ability to withstand load and provides lubricating ability. Table 4 illustrates that hardness increases significantly for DLC-coated dimpled surfaces. Previous studies also showed that DLC coating produced a high hardness [23, 33]. However, the Vickers hardness number was lower near the dimple area (T_2_), which can be attributed to the geometrical effect of the dimple and the changes in microstructure during dimple fabrication. However, the average value of the three positions was nearly the same as the plain surface (T_1_).

The residual stress was measured to identify the stresses that persist in a solid material after the original cause of the stress is removed. The residual stress was focused on the dimple area. The high value of residual stress confirms the poor surface adhesion of the DLC coating, which can cause early delamination of coated materials during friction at high loads.

### 3.4. Wettability

Table 4 clearly illustrates the variation in contact angle for different surface conditions over different lubricants. Prior to the experiment, different lubricants were used to find their interaction with solid materials. A DLC-coated surface is considered mildly hydrophobic [33, 34]. In our studies, similar trends are found. The contact angle for the T_3_ surface has been increased compared to the T_1_ and T_2_ surfaces over all lubricants. The increase in contact angle confirms the reduction of surface energy. As a result, the coated surface provides less attraction to water particles and thus helps in protein adsorption. Protein can form a stable layer that enhances lubricating ability.

Moreover, the contact angle for the OASF lubricant on T_3_ is higher (72 ± 3°) than the BS lubricant (57 ± 3°), and this confirms that the hydrophobic nature of the DLC-coated surface is more intense for the OASF lubricant than BS, and so results in a better interaction of fluid particles with coated surfaces. Furthermore, OASF has a wider range of protein composition than BS, and, eventually, it reduces the friction coefficient during friction testing.

### 3.5. Friction tests

#### 3.5.1. Effects of surface conditions

**Figure 3.**
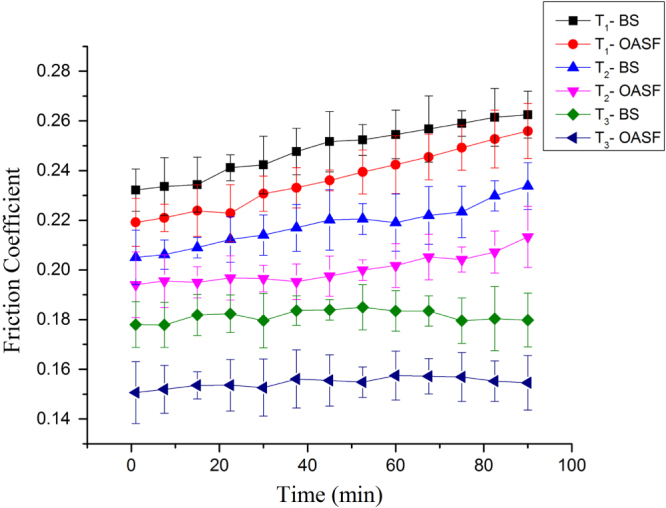
Friction coefficient profiles over time at 131 MPa under BS and OASF lubricants.

The friction coefficient is considered a key parameter for tribological outputs, where a reduction in friction value is desirable to reduce wear of a joint prosthesis. The change in friction coefficient value with time under different lubrication condition of BS and OASF is presented in figure 3, which identifies the tribological effect of lubricants with three different surface conditions. It shows that OASF lubricants provide the lowest friction coefficient (0.157) for the T_3_ surface, and BS gives the highest friction coefficient (0.248) for the T_1_ surface under the same contact pressure of 131 MPa.

It is easily understood from figure 3 that OASF exhibits a comparatively lower friction coefficient than BS lubricants, which confirms the better lubricating abilities of OASF for all surface conditions. However, the trend of the friction coefficient profile over time for the same experimental conditions stated in table 2 are quite stable for T_3_ surfaces, while the other shows increasing order with increasing time. The stability of the friction value for the T_3_ surface may be attributed to the improved mechanical and surface properties (details are in tables 4 and 5). In addition, there are statistically significant differences (*p* < 0.05) in friction coefficient values over time between the groups, and they are homogenous (*p* > 0.05) across each group for the same loading conditions.

**Table 5.** Contact angle on different surface conditions before friction tests.

	Contact angle for different lubricants (°)		
Surface condition	*θ*_water_	*θ*_BS_	*θ*_OASF_
Plain surface (T_1_)	65 ± 3	35 ± 3	50 ± 3
Dimpled surface (T_2_)	70 ± 4	38 ± 4	52 ± 4
DLC-coated dimpled surface (T_3_)	87 ± 3	57 ± 3	72 ± 3

Lubricant makes the difference in the friction coefficient value because the composition of the lubricant plays a significant role in reducing friction. Generally, fetal BS contains 20–36 mg ml^−1^ albumin, which is also present in normal synovial fluid (SF) in a range of 11.65–12.92 mg ml^−1^, while OA patients carry 17.75–18.45 mg ml^−1^ albumin [25]. Moreover, albumin is considered an important biological component that helps in boundary lubrication through adsorption to joint materials and often provides a soft boundary layer between the contact surfaces. BS does not have other protein compositions (globulin and lubricin) and HA, which are important for lubrication; however, OASF contains important biological components such as globulin, lubricin, and HA, along with albumin, that help in lubrication and thus provide the lower friction coefficient. HA increases the viscosity of a lubricant and thus helps in better lubrication [35]. Other protein components also are relevant; thus, globulin helps in artificial joints by blocking metal ion removal from the surface layers, and lubricin reduces the shear strength at the asperity contact interface [25]. HA plays a major role in lubrication, similar to the role of additives in commercial lubrication, and enhances the lubricating ability of these protein components [25]. Thus it is considered a key component of SF to reduce friction and wear. We found viscosity 2.58 cP and 1.34 cP for the lubricant OASF and BS, respectively. It confirms that HA increases the viscosity of OASF significantly as compared with the viscosity of BS. The higher viscosities of OASF reduce the friction between contact surfaces by preventing two surfaces coming in contact. In previous studies, it was found that lubricant containing albumin, globulin, HA, and lubricin provided the lowest friction coefficient, and this is similar to our experimental results [25]. OASF along with DLC coating exhibits the lowest friction coefficient, confirming the better interaction of the DLC-coated surface with the biological components of SF.

More specifically, T_2_ and T_3_ surfaces reduce the friction coefficient by 16% and 33%, respectively, more than that of the T_1_ surface under OASF lubricant at the same contact pressure of 131 MPa. In addition, similar trends are found for BS lubricants, where the T_2_ and T_3_ surfaces decreased the friction coefficient by 10% and 26% more than that of the T_1_ surface, respectively. It confirms the better lubricating ability of the DLC-coated dimpled surface. It was reported in previous studies that a dimpled surface reduces the friction because it increases hydrodynamic pressure under sliding conditions [12, 36, 37]. Our study shows similar results. The frictional behavior of micro-dimples may also be attributed to the decreased contact area and the dimples supplying lubricant continuously in the contact region. Micro-dimples can improve anti-seizing ability by reserving lubricant, improve additional lift by generating hydrodynamic pressure, reduce adhesion by reducing contact area, and also prevent abrasive wear by trapping wear debris generated by friction [12]. In our studies, it was found that the DLC-coated dimpled surface enhanced the lubricating ability and, thus, reduced friction between the contact surfaces. The low friction coefficient of the T_3_ surface can be attributed to its higher hardness. Moreover, it forms a graphite transfer film on the counter surface, due to the graphitization of coated materials, which helps provide easy slip between contact surfaces and thus minimizes friction.

#### 3.5.2. Effects of different loads

**Figure 4.**
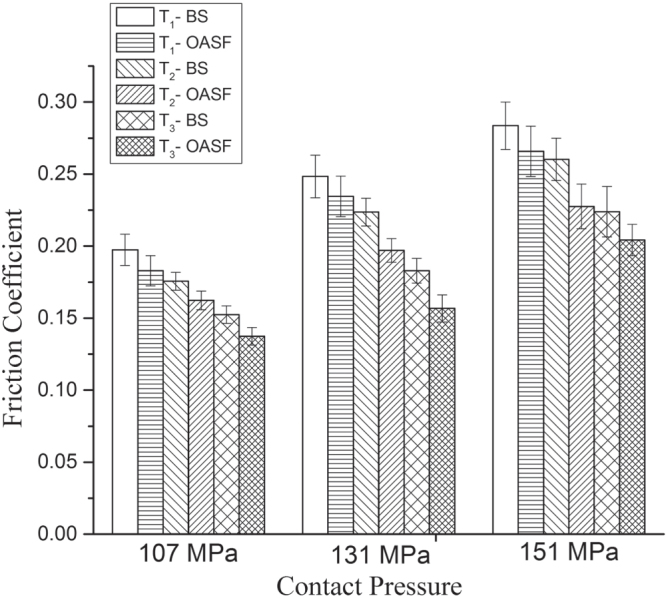
Average friction coefficient at contact pressure for BS and OASF lubricants.

The friction test was conducted on three different loads, stated in table 2. Experimental conditions were similar to body movement that represents medium walking conditions for hip joints. The friction coefficient profiles were obtained at contact pressures of 107, 131, and 151 MPa, respectively, applying normal loads of 10, 15, and 20 N. The friction profiles for the three loading conditions are presented in figure 4. It clearly demonstrates that friction coefficients increase with increasing load. The friction coefficient increases 26%, 27%, and 20%, respectively, for T_1_, T_2_, and T_3_ surfaces as a result of increasing loads of 107–131 MPa in the presence of the same lubricant, BS. With increasing load, the contact area may increase, and the dimpled surfaces are not able to bear high load. As a result, the increasing rate of the friction value for the dimpled surface is quite higher than the plain surface. The lower increasing rate of friction value for the T_3_ surface with increasing load may be attributed to its high hardness value. Furthermore, DLC enhances the lubricating ability due to its solid lubricant activity during sliding. In addition, a similar phenomenon is found under OASF lubricants. Thus, DLC coating improves the lubricating ability on dimpled interfaces.

Clearly, for the T_3_ surface, with increasing load, the graphitization of coated material increases and so reduces the rate of increasing friction value. However, the friction coefficient value increases dramatically when the load increases to 151 MPa under different lubrication conditions. This happens because of full delamination of coated materials under high load. As the graphitization is directly proportional to load, at a certain high load the coated materials split out from the surfaces. Still, OASF lubricants provides a comparatively lower friction coefficient value compared to BS and prove the load-withstanding ability of OASF is better than BS when it acts as a lubricant between the tribopairs.

### 3.6. Raman analysis

**Figure 5.**
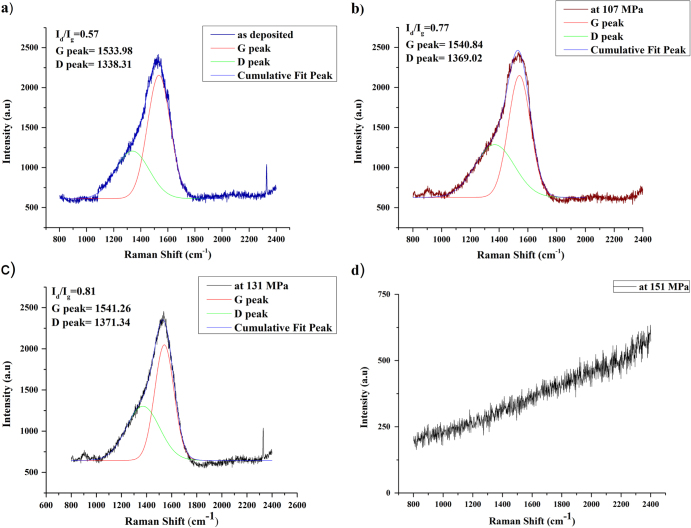
Raman analysis of DLC-coated sample tested at different load conditions.

DLC is a combination of sp^2^ and sp^3^ hybridized bonds, where tribological outcomes of these coatings depend on the sp^2^/sp^3^ bonding ratio [23, 38]. The relative ratio of the D peak and G peak (*I*_d_/*I*_g_) plays a significant role in Raman spectra to characterize the sp^2^/sp^3^ bonding ratio [23]. The sp^2^/sp^3^ bonding ratio is also considered a dominant factor in determining the quality of DLC-coated surfaces. Both peaks are decorated after the Gaussian fitting method. The Raman analysis results of the T_3_ surfaces under different loading conditions are presented in figure 5. There is a significant change in the *I*_d_/*I*_g_ ratio when the load is applied. Under a deposited condition, the *I*_d_/*I*_g_ ratio was 0.57. As the sample goes through the loading condition, the *I*_d_/*I*_g_ ratio increases.

While the sample was tested at deposited and at 107 MPa load, the *I*_d_/*I*_g_ ratio changed from 0.57 to 0.77. This confirms the graphitization of the coated materials. Similar trends of increasing *I*_d_/*I*_g_ ratio are found in previous studies that revealed that the *I*_d_/*I*_g_ ratio increases at deposited condition to the wear track region [8, 23, 39]. It indicates a poor quality of DLC coating in the wear track region, identified by a higher value in the sp^2^/sp^3^ or *I*_d_/*I*_g_ ratio. Moreover, hardness and modulus of elasticity vary, depending on these sp^2^/sp^3^ bonding ratios [8]. In addition, G peaks and D peaks move to higher wave numbers with an increasing load, possibly due to the microstructural modification in DLC film during sliding of friction tests. Also, with a change in load from 107 MPa to 131 MPa, the *I*_d_/*I*_g_ increases from 0.77 to 0.81. Although the change in peak wave number is significant from the deposited condition to 107 MPa, there is a smaller change with an increase in load from 131 MPa to 151 MPa. Moreover, peak intensity also increases with increasing load.

Notably, there is a significant correlation between friction coefficient value and *I*_d_/*I*_g_ ratio. The smaller increase in the *I*_d_/*I*_g_ ratio increases the graphitization of the coated material. Hence, the surface becomes smoother when a transfer of graphite film forms on the counter surface. The friction coefficient value decreases due to graphitization with increasing *I*_d_/*I*_g_ ratio. The *I*_d_/*I*_g_ ratio is high (0.81) at 131 MPa, which confirms a high degree of graphitization. Graphite-like sp2 type content has a low shear strength, and, hence, coated materials are progressively removed from the surface [38]. Interestingly, the friction coefficient increases rapidly over the all lubricants at 151 MPa, because the transfer film also moves from the contact area due to higher load. Furthermore, no peak can be observed in the scanning region of the Raman spectra (figure 5(d)), as the coated materials completely split out at higher loads.

### 3.7. Wear analysis

**Figure 6.**
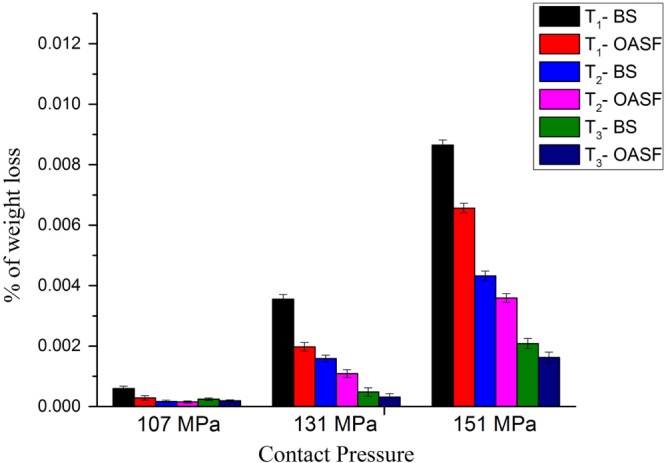
Weight loss of disk materials after friction test.

A low wear rate is desirable for an artificial joint prosthesis, as a high wear rate decreases its useful life. Researchers have identified a correlation between friction coefficient and wear rate [27, 31], and in previous studies, it has been found that wear rate increases with increasing friction coefficient over different types of lubricants [12]. Our experimental results demonstrate similar trends. The wear rate was calculated by the weight loss of the disk material both before and after testing. The weight loss at 107 MPa was negligible; nevertheless, the weight loss became high for plain surface conditions over all lubricants and with different loads. The weight losses of the disk material at three different test loads are presented in figure 6.

However, the lower wear rate for T_3_ surfaces compared to those of T_1_ and T_2_ is associated with a low friction coefficient. The reason of weight loss for T_3_ at higher loads can be explained by the graphitization of the coated materials. Nevertheless, the weight loss of disk material is very high at 151 MPa, whatever the lubricant in the friction test. It confirms that lubricant-like body fluids cannot withstand high load. Although the weight loss for OASF lubricant is lower than for BS lubricant, it shows a higher wear rate for the T_1_ and T_2_ surfaces, which may be attributed to the poor load-withstanding ability of the surface at higher loads, as well as the hardness of the material. The wear resistance depends on the ratio of hardness (H) and modulus of elasticity (E). Previous studies also reveal that a high value of H/E exhibits high wear resistance of the material [8]. In our study, a lower wear rate for the T_3_ surface can be explained by an H/E ratio compared to the T_1_ and T_2_ surfaces.

**Figure 7.**
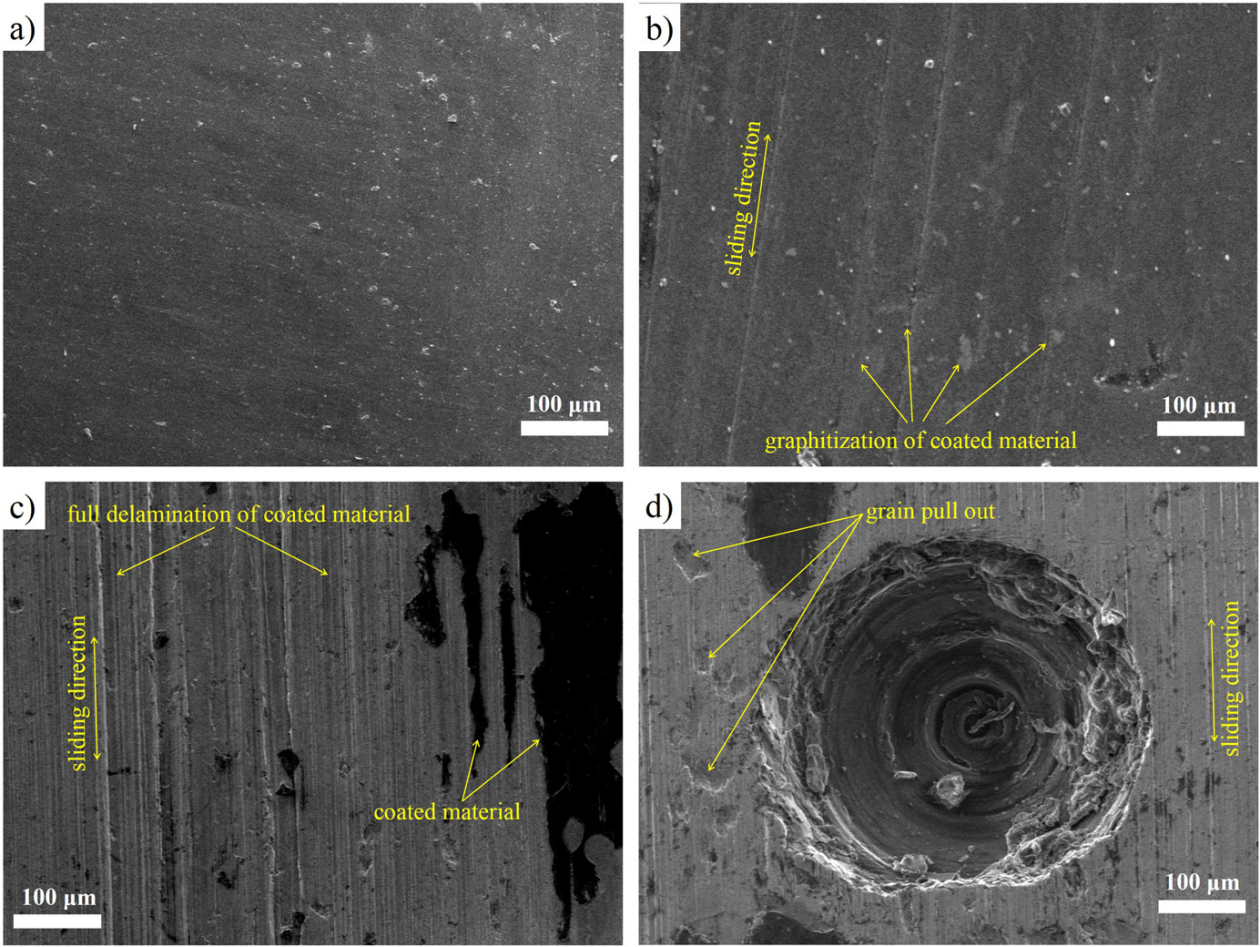
FESEM images showing (a) as-deposited DLC-coated surface, (b) formation of film transfer due to load, (c) full delamination of coated materials at higher loads, and (d) wear track on dimpled area.

However, the FESEM image also reveals the true morphology of the worn surfaces. It was quite impossible to distinguish the worn surfaces for the same load with different lubricants. The surface morphology due to applied different loads is demonstrated in figure 7. Figure 7(a) describes the deposited condition of the surface (T_3_), and figure 7(b) describes the surface at applied load condition. The graphitization of the coated material happened with the applied load and coated materials transferred from the surface at a lesser rate at low load (figure 7(b)), but it increases rapidly at higher loads. Previous studies also reported that the formation of film transfer occurs during sliding contact of materials [23, 40]. Figure 7(c) shows coated materials are fully delaminated from the contact region due to higher loads. It confirms that the DLC-coated sample can bear the loads only up to a certain limit. After that, it pulls out from the surface with an increasing transfer film formation rate. Furthermore, figure 7(d) shows that there is damage to the structure of the dimple at higher loads, which may be attributed to the lower hardness of the titanium alloy. Also, there is some grain pull-out during sliding contact at higher loads, as also seen in previous studies [14].

This study has been conducted based on the friction coefficient and wear rate of joint implants. Further investigation on film formation of lubricant and its adsorption and desorption on coated surfaces would suggest guidelines for the development of optimized surfaces with better control of friction and wear in joint implants.

## 4. Conclusions

The tribological performance of advanced interfaces, namely, dimpled and DLC-coated dimpled surfaces, was investigated under a formulated OASF and BS, using a pin-on-disk tribometer. The comparison between different surface conditions revealed that the maximum reduction of friction coefficient was 33% by a DLC-coated dimpled surface, whereas 16% by a dimpled surface, compared to a plain surface for 131 MPa contact pressure under OASF condition. The effects of lubricant viscosity and surface wettability were found to be significant on tribological performance. The hardness and residual stress exhibited maxima at 698 HV and 477 MPa, respectively, for a DLC-coated dimpled surface; hence, it lowered the friction and wear rate. The friction coefficient increased with increasing load; however, the rate of increase was lower for DLC-coated dimpled surfaces due to the graphitization of the coated materials. The formation of transfer graphite film was confirmed by Raman spectroscopy. Overall, considering friction coefficient and wear rate, it can be concluded that the DLC-coated dimpled surface would be beneficial in artificial joint implants for OA patients.

## References

[C1] Blagosklonny M V (2010). Why human lifespan is rapidly increasing: solving ‘longevity riddle’ with ‘revealed-slow-aging’ hypothesis. Aging.

[C2] Harris W H (1995). The problem is osteolysis. Clin. Orthopaed. Relat. Res..

[C3] Choudhury D, Urban F, Vrbka M, Hartl M, Krupka I (2015). A novel tribological study on DLC-coated micro-dimpled orthopedics implant interface. J. Mechan. Behavior Biomed. Mat..

[C4] Liu F, Jin Z, Roberts P, Grigoris P (2006). Importance of head diameter, clearance, and cup wall thickness in elastohydrodynamic lubrication analysis of metal-on-metal hip resurfacing prostheses. Proc. Inst. Mechan. Eng. H: J. Engin. Med..

[C5] Choudhury D, Ay Ching H, Mamat A B, Cizek J, Osman A, Azuan N, Vrbka M, Hartl M, Krupka I (2014). Fabrication and characterization of DLC coated microdimples on hip prosthesis heads. J. Biomed. Mater. Res. B: Appl. Biomater..

[C6] Wimmer M, Loos J, Nassutt R, Heitkemper M, Fischer A (2001). The acting wear mechanisms on metal-on-metal hip joint bearings: *in vitro* results. Wear.

[C7] Firkins P, Tipper J, Saadatzadeh M, Ingham E, Stone M, Farrar R, Fisher J (2001). Quantitative analysis of wear and wear debris from metal-on-metal hip prostheses tested in a physiological hip joint simulator. Bio-Med. Mater. Eng..

[C8] Amanov A, Sasaki S (2013). A study on the tribological characteristics of duplex-treated Ti–6Al–4V alloy under oil-lubricated sliding conditions. Tribol. Int..

[C9] Amanov A, Cho I-S, Kim D-E, Pyun Y-S (2012). Fretting wear and friction reduction of CP titanium and Ti–6Al–4V alloy by ultrasonic nanocrystalline surface modification. Surf. Coat. Technol..

[C10] Masmoudi M, Assoul M, Wery M, Abdelhedi R, El Halouani F, Monteil G (2006). Friction and wear behaviour of cp Ti and Ti6Al4V following nitric acid passivation. Appl. Surf. Sci..

[C11] Qiu Y, Khonsari M (2011). Experimental investigation of tribological performance of laser textured stainless steel rings. Tribol. Int..

[C12] Roy T, Choudhury D, Bin Mamat A, Pingguan-Murphy B (2014). Fabrication and characterization of micro-dimple array on Al_2_O_3_ surfaces by using a micro-tooling. Ceram. Int..

[C13] Sawano H, Warisawa S I, Ishihara S (2009). Study on long life of artificial joints by investigating optimal sliding surface geometry for improvement in wear resistance. Precision Engin..

[C14] Roy T, Choudhury D, Ghosh S, Mamat A B, Pingguan-Murphy B (2015). Improved friction and wear performance of micro dimpled ceramic-on-ceramic interface for hip joint arthroplasty. Ceram. Int..

[C15] Hauert R (2003). A review of modified DLC coatings for biological applications. Diam. Relat. Mater..

[C16] Meletis E, Erdemir A, Fenske G (1995). Tribological characteristics of DLC films and duplex plasma nitriding/DLC coating treatments. Surf. Coat. Technol..

[C17] Field S, Jarratt M, Teer D (2004). Tribological properties of graphite-like and diamond-like carbon coatings. Tribol. Int..

[C18] Manhabosco T, Barboza A, Batista R, Neves B, Müller I (2013). Corrosion, wear and wear–corrosion behavior of graphite-like *a*-C: H films deposited on bare and nitrided titanium alloy. Diam. Relat. Mater..

[C19] Heuberger M P, Widmer M R, Zobeley E, Glockshuber R, Spencer N D (2005). Protein-mediated boundary lubrication in arthroplasty. Biomaterials.

[C20] Wang A, Essner A, Schmidig G (2004). The effects of lubricant composition on *in vitro* wear testing of polymeric acetabular components. J. Biomed. Mater. Res.—Part B Appl. Biomater..

[C21] Myant C, Underwood R, Fan J, Cann P M (2012). Lubrication of metal-on-metal hip joints: The effect of protein content and load on film formation and wear. J. Mechan. Behavior Biomed. Mater..

[C22] Vrbka M, Křupka I, Hartl M and Návrat T 2014 *In situ* measurements of thin films in bovine serum lubricated contacts using optical interferometry Proc. Inst. Mechan. Eng. H: J. Engin. Med. 228 149 58 149–58 0954411913517498 10.1177/0954411913517498 24398447

[C23] Al Mahmud K, Varman M, Kalam M, Masjuki H, Mobarak H, Zulkifli N (2014). Tribological characteristics of amorphous hydrogenated (aC: H) and tetrahedral (ta-C) diamond-like carbon coating at different test temperatures in the presence of commercial lubricating oil. Surf. Coat. Technol..

[C24] Chan S M T, Neu C P, Komvopoulos K, Reddi A H (2011). The role of lubricant entrapment at biological interfaces: reduction of friction and adhesion in articular cartilage. J. Biomech..

[C25] Ghosh S, Choudhury D, Das N S, Pingguan-Murphy B (2014). Tribological role of synovial fluid compositions on artificial joints—a systematic review of the last 10 years. Lubricat. Sci..

[C26] Hodge W, Fijan R, Carlson K, Burgess R, Harris W, Mann R (1986). Contact pressures in the human hip joint measured *in vivo*.

[C27] Mischler S, Munoz A I (2013). Wear of CoCrMo alloys used in metal-on-metal hip joints: a tribocorrosion appraisal. Wear.

[C28] Spriano S, Vernè E, Faga M, Bugliosi S, Maina G (2005). Surface treatment on an implant cobalt alloy for high biocompatibility and wear resistance. Wear.

[C29] Shroder R, Nemanich R, Glass J (1990). Analysis of the composite structures in diamond thin films by Raman spectroscopy. Phys. Rev. B.

[C30] Meng F, Davis T, Cao J, Wang Q J, Hua D, Liu J (2010). Study on effect of dimples on friction of parallel surfaces under different sliding conditions. Appl. Surf. Sci..

[C31] Gispert M, Serro A, Colaco R, Saramago B (2006). Friction and wear mechanisms in hip prosthesis: comparison of joint materials behaviour in several lubricants. Wear.

[C32] Sheeja D, Tay B, Lau S, Nung L (2001). Tribological characterisation of diamond-like carbon coatings on Co–Cr–Mo alloy for orthopaedic applications. Surf. Coat. Technol..

[C33] Ching H A, Choudhury D, Nine M J, Osman N A A (2014). Effects of surface coating on reducing friction and wear of orthopaedic implants. Sci. Technol. Adv. Mater..

[C34] Chen S-Y, Ou K-L, Huang W-C, Chu K-T, Ou S-F (2013). Phase transformation of diamond-like carbon/silver composite films by sputtering deposition. Ceram. Int..

[C35] Trunfio-Sfarghiu A M, Berthier Y, Meurisse M H, Rieu J P (2007). Multiscale analysis of the tribological role of the molecular assemblies of synovial fluid. Case of a healthy joint and implants. Tribol. Int..

[C36] Choudhury D, Walker R, Roy T, Paul S, Mootanah R (2013). Performance of honed surface profiles to artificial hip joints: an experimental investigation. Int. J. Precision Engin. Manufact..

[C37] Choudhury D, Walker R, Shirvani A, Mootanah R (2013). The influence of honed surfaces on metal-on-metal hip joints. Tribol. Online.

[C38] Jaoul C, Jarry O, Tristant P, Merle-Mejean T, Colas M, Dublanche-Tixier C, Jacquet J-M (2009). Raman analysis of DLC coated engine components with complex shape: understanding wear mechanisms. Thin Solid Films.

[C39] Liu Y, Erdemir A, Meletis E (1996). A study of the wear mechanism of diamond-like carbon films. Surf. Coat. Technol..

[C40] Rigney D, Chen L, Naylor M G, Rosenfield A (1984). Wear processes in sliding systems. Wear.

